# Milk Thistle Oilseed Cake Flour Fractions: A Source of Silymarin and Macronutrients for Gluten-Free Bread

**DOI:** 10.3390/antiox11102022

**Published:** 2022-10-13

**Authors:** Jan Bedrníček, František Lorenc, Markéta Jarošová, Veronika Bártová, Pavel Smetana, Jaromír Kadlec, Dana Jirotková, Jan Kyselka, Eva Petrášková, Marie Bjelková, Petr Konvalina, Trong Nghia Hoang, Jan Bárta

**Affiliations:** 1 Department of Food Biotechnologies and Agricultural Products’ Quality, Faculty of Agriculture and Technology, University of South Bohemia in České Budějovice, Studentská 1668, 370 05 České Budějovice, Czech Republic; 2 Department of Plant Production, Faculty of Agriculture and Technology, University of South Bohemia in České Budějovice, Na Sádkách 1780, 370 05 České Budějovice, Czech Republic; 3 Department of Dairy, Fat and Cosmetics, Faculty of Food and Biochemical Technology Prague, University of Chemistry and Technology, Technická 5, 166 28 Prague, Czech Republic; 4 Department of Animal Husbandry Sciences, Faculty of Agriculture and Technology, University of South Bohemia in České Budějovice, Studentská 1668, 370 05 České Budějovice, Czech Republic; 5 Department of Legumes and Technical Crops, Agritec Plant Research, Ltd. Zemědělská 2520, 787 01 Šumperk, Czech Republic; 6 Department of Agroecosystems, Faculty of Agriculture and Technology, University of South Bohemia in České Budějovice, Branišovská 1645, 370 05 České Budějovice, Czech Republic

**Keywords:** gluten-free bread, oilseed cake flour, dry sieving, baking, dough rising, silymarin stability, sensory analysis, nutrition profile

## Abstract

The utilization of plant by-products as functional food ingredients has received increasing attention in the last decade. One such by-product generated during milk thistle oil pressing is oilseed cakes, which could be used as a novel food ingredient. Therefore, the study aimed at investigating the effects of the addition of milk thistle oilseed cake (MTOC) flour fractions obtained via dry sieving, differing in particle size (unsieved; coarse: >710 µm; medium: 315–710 µm; and fine: <315 µm), on the quality of gluten-free bread and stability of silymarin during breadmaking. The 10% addition of the fractions into gluten-free bread increased the protein, fibre, fat, ash and silymarin content. The breads with the coarse fraction had the highest content of fibre, whereas the breads with the fine fraction excelled in protein, fat and ash content. The medium fraction was characterized as the richest source of silymarin, whilst the fine fraction was the poorest. Silymarin constituents were slightly released during dough rising but also partially decomposed during baking; moreover, silydianin was the most susceptible and degraded the most. The enriched breads had better sensory and textural properties compared to the control bread. The results suggest that MTOC flour fractions can improve the potential health benefits and nutritional profile of gluten-free bread.

## 1. Introduction

People suffering from coeliac disease strictly adhere to a gluten-free (GF) diet since it is the only treatment. Unfortunately, this diet may represent a risk for consumers to some extent because it has been reported that it is characterized by a lower content of fibre and protein, on the one hand, and by an excessive content of fat and carbohydrates such as starch, on the other hand [1,2,3].

Gluten, the protein triggering typical symptoms of coeliac disease, which must be omitted in a GF diet, also plays an important technological role due to its viscoelastic properties that are responsible for the palatability, crunchiness and structural integrity of bakery products. Replacing gluten-containing flour with GF flour makes the resulting bakery product (especially bread) less sensorially attractive. For example, such bread has a crumbly texture and goes stale faster. The development of GF products is, however, a technological challenge for the food industry [4]. To avoid these problems, producers of GF bread usually add additives that improve texture properties to mitigate the loss of gluten [5].

Coeliac disease, thus, has led scientists to search for GF alternatives to wheat flour for bakery products. As reported by Mir et al. [6], researchers have taken different approaches, such as the use of GF cereal and pseudocereal flours (rice, maize, sorghum, quinoa, amarant, or buckwheat), legume flours (soya, chickpea, or pea), starches (corn, potato, or cassava) and additives such as hydrocolloids (e.g., certain polysaccharides and proteins), and shortenings, or combinations of the above-mentioned, to improve technological, sensory and nutritional properties together with shelf-life. The use of enzymes and sourdough is also an option for the development of good-quality GF bread [7]. One of the promising ingredients that possess interesting functional properties is oilseed cakes generated after oil pressing from oil crops [8]. Although these by-products can contain a wide variety of nutrients (e.g., proteins, polysaccharides and/or unsaturated fatty acids) and biologically-active compounds with potential health benefits for humans (e.g., polyphenols), the latter-mentioned authors add that this type of by-product is mostly used as feed. For such reasons, scientists have tried to valorise these by-products for human nutritional purposes, which also supports the idea of the circular economy. Moreover, the utilization of oilseed cakes other than into bakery products is well documented in original papers, e.g., Guo et al. [9], or a review by Nevara et al. [10]. Information about the incorporation of oilseed cakes into GF bakery products is, however, very limited.

A very interesting GF oil crop is milk thistle (*Silybum marianum* L.), which is well known for its nutritious oil and presence of silymarin, a complex of polyphenolic compounds composed of six main flavonolignans (silychristin, silydianin, silybin A, silybin B, isosilybin A and isosilybin B) and taxifolin, which belongs to the polyphenolic group of flavanonols [11,12]. Bárta et al. [8] reported that milk thistle oilseed cake (MTOC) flour contains approximately 22% crude protein, 5% crude fat, 64% carbohydrates and 6% ash. From this point of view, milk thistle seems to be a promising ingredient for GF bakery products since it contains a relatively high amount of protein and carbohydrates with interesting functional properties. At the same time, the sensory properties of GF bread, which might change after the addition of MTOC flour, must not be neglected. For example, flaxseed oilseed cakes added into the bread in the amount of 5 and 7.5% significantly increased consumers’ acceptance [13].

Furthermore, any flour, including one produced from oilseed cakes, can be fractionalized using sieves with a different mesh size to obtain flour fractions with altered nutritious and technological properties [14]. In particular, authors have stated that the smaller the flour particle size, the higher the content of protein, fat and ash (as a part of endosperm) and the lower content of fibre, and vice versa. For instance, Stevenson et al. [15] sieved oat pin-milled flour and found that the β-glucan content in fractions with a particle size of 300–850 µm, 150–300 µm and <150 µm decreased from 4.2 to 2.3 and 0.8%, respectively. According to Protonotariou et al. [16], sieving wheat flour into particles with a size of <100 µm substantially increases water-holding capacity, oil-holding capacity, and ash and protein content compared to fractions with particle sizes of >200 µm. Nevertheless, there is no published paper focusing on the addition of MTOC flour fractions into GF bread.

Regarding the fortification of GF bread with MTOC flour fractions, it should be noted that coeliac disease is often linked with liver disease and dysfunctions such as hypertransaminasemia, autoimmune hepatitis and cirrhosis [17,18], and, as mentioned before, milk thistle contains silymarin complex that has been used for decades as a hepatoprotective drug with scientifically-proven positive effects in several clinical trials [19]. In most of these clinical trials, silymarin was used as milk thistle achenes extract in the form of capsules or tablets. Moreover, silymarin complex exerts anti-inflammatory, antioxidant, antibacterial, antiallergic and antimutagenic properties, and lowers blood glucose levels [19,20].

Therefore, the incorporation of MTOC flour fractions into GF bread might be beneficial from three aspects; it improves its nutrition profile, enhances its therapeutic potential, and, also, oilseed cakes, considered as a waste or by-product, will be directly used for food production.

However, before testing the therapeutic potential (e.g., in clinical trials) of GF bakery products containing silymarin in its non-extracted form, such as oilseed cakes flour fractions, it must be tested whether the enriched GF bread is acceptable for consumers and if silymarin is thermally stable and will remain in baked bread, and, thus, what will be the final content in the bread. It was reported that some of the polyphenolic compounds are sensitive to food processing, mainly to the high temperatures used during baking, which usually lowers their content and alters antioxidant activity–although the rupture of cell walls due to heat can improve mobility and their bioavailability [21]. Polyphenols can also be positively affected by fermentation processes, such as the rising of the dough, because they can be released from bonds with polysaccharides and can be easily extracted and more bioavailable [22]. Conversely, Lukšič et al. [23] reported that rutin was degraded to quercetin during Tartary buckwheat sourdough fermentation, but the quercetin was thermally stable to some extent and was detected in baked bread. Nevertheless, to date, the influence of food processing on silymarin quantity and quality has not been published.

Considering all the facts given above, the aims of the study were:

i. To assess the effect of the addition of different MTOC flour size fractions into GF bread on the selected qualitative parameters; and,
ii. To investigate the effects of bread processing (dough rising and baking) on the antioxidant activity and content of silymarin in GF breads.

The study provides information on whether MTOC flour size fractions can be used as an ingredient suitable for GF bread with potentially elevated health benefits.

## 2. Materials and Methods

### 2.1. Milk Thistle Oilseed Cake Flour Preparation

Milk thistle seeds of the Mirel variety, provided by Agritec Plant Research Ltd., were conditioned on a Stephan UM/SK 5 industrial unit (Stephan Machinery, Hameln, Germany), equipped with a 5-litre duplicated stainless steel vessel. Conditioning was done by direct saturated steam injection (2 bar) into the duplicated vessel for 30 s while stirring the seeds with a Teflon wiper. Afterwards, the conditioned material was screw-pressed by a Komet CA59G oil expeller (IBG Monforts, Oekotec GmbH, Mönchengladbach, Germany) with an optimal worm shaft frequency of 55 RPM. A nozzle with a diameter of 8 mm was used at the outlet of the screw-pressing unit.

The prepared oilseed cakes were then disintegrated and milled using a Grindomix GM 200 knife mill (Retsch, Haan, Germany) at 3000 RPM for 2 min. The resulting homogenised material was assigned as unsieved and was subsequently separated into fractions differing in particle size using AS 200 analytical vibrating sieves (Retsch, Haan, Germany): coarse (>710 µm), medium (315–710 µm) and fine (<315 µm). Thus, four MTOC flour fractions in total were obtained: unsieved, coarse, medium and fine. Afterwards, all the obtained flour fractions were standardised to the same particle size using a hammer mill equipped with a 60-mesh sieve. Thus, very fine flour fractions were obtained. The reason for the particle size standardisation step was that it would otherwise be impossible to incorporate the coarse fraction into the bread due to the very big pieces and very hard texture of milk thistle achenes. All the flour fractions were then used for the fortification of GF bread and chemical analyses: basic chemical composition, analysis of silymarin and antioxidant activity. Before the analyses were conducted, the flour fractions were lyophilised. The flour fractions’ characteristics are shown in Table 1.

### 2.2. Bread Baking and Sample Collection

MTOC flour fractions were incorporated into the GF control bread based on a recipe that was composed of the following ingredients (all percentages are *w*/*w*): sorghum flour 23.20%, corn flour 11.60%, corn starch 5.80%, potato starch 5.80%, tapioca starch 5.80%, guar flour 0.52%, sunflower oil 2.32%, salt 0.81%, dried yeasts (*S. cerevisiae*) 0.58% and water 43.56%. In experimental batches, 10% of the GF flour blend was replaced by the unsieved, coarse, medium or fine MTOC flour fractions. The amount of added oil, salt, yeasts and water remained unchanged. The detailed recipe is shown in Supplementary Materials Table S1. Thus, five groups of GF breads were made: control, unsieved, coarse, medium and fine. The amount of replaced GF flour blend was based on a pilot experiment and the addition of 10% was evaluated as the best mainly based on sensory properties and the specific volume of the bread loaf (data not given).

The breadmaking was divided into three steps. First, all ingredients were gently mixed in a kitchen robot for 20 min to make a dough. Second, portions of the dough (400 g) were filled into cuboid baking forms (three forms per group) and the dough was allowed to rise at 33 °C for 60 min. The final step was the baking of the bread at 170 °C for 45 min. The process of rising and baking was conducted in a combi oven (Convotherm 4EasyTouch 6.10EB, Welbilt Deutschland GmbH, Eglfing, Germany). Three loaves of bread were made for each group.

To determine the effect of dough rising and the baking of the bread on the concentration of silymarin complex, and antioxidant properties, samples of the dough were taken before rising, after rising and after baking.

Afterwards, all dough and bread samples intended for chemical analyses (basic chemical composition, analysis of silymarin complex and antioxidant activity) were immediately put into a freezer at −18 °C and then lyophilised and stored at −18 °C until analysis. Fresh bread samples were used for the analyses of specific loaf volume, colour and texture, and also for sensory analysis.

### 2.3. Chemical Analyses of the Doughs, Breads and Milk Thistle Oilseed Cake Flour Fractions

#### 2.3.1. Basic Chemical Composition

The content of crude protein, crude fat, ash, water and non-soluble fibre fractions were analysed in the bread samples and flour fractions.

The crude protein content of the samples was measured using a Rapid N Cube elementary analyser (Elementar AnalysenSysteme, Langenselbold, Germany) based on a modified combustion Dumas method according to the manufacturer’s instructions. The crude protein content was calculated as nitrogen content multiplied by a factor of 6.25 [8].

The crude lipid content was determined using an ANKOM XT10 extractor (Ankom Technology, Macedon, NY, USA) with petroleum ether as a solvent. Firstly, samples (1 g) were placed in filter bags. Then, petroleum ether extraction was conducted using the extractor. The fat content was calculated as the relative difference in weight of the bag containing the sample before and after the extraction [8].

The moisture content was analysed by a gravimetric method by drying the sample at 105 °C until constant weight. The ash content was also gravimetrically determined by combusting the sample at 550 °C for 16 h in a muffle furnace to obtain a light grey ash [24].

The neutral detergent fibre (NDF), acid detergent fibre (ADF) and acid detergent lignin (ADL) were analysed according to the modified method of Van Soest et al. [25] using a FT 122 Fibertec fibre analyser (Foss, Hilleroed, Denmark). For the NDF analysis, the sample was hydrolysed in a neutral detergent agent (sodium lauryl sulphate) using the fibre analyser. The NDF was calculated as the difference in the weight before and after the neutral hydrolysis. The ADF content was gravimetrically determined after acid hydrolysis (0.5 M sulfuric acid) of the sample in the presence of cetyltrimethylammonium bromide in the fibre analyser. Gravimetric determination of ADL in the samples was determined after acid hydrolysis (72% sulfuric acid) using the abovementioned fibre analyser.

Based on these analyses, the cellulose, hemicellulose and nitrogen-free extract (NFE) content were calculated as follows:

cellulose = NDF−ADF

hemicellulose = ADF−ADL,

NFE = 100 - (% of water, ash, crude protein, cellulose, hemicellulose, lignin and crude fat).

The total energy (kJ/100 g) of the bread samples and flour fractions was calculated using conversion factors according to Regulation (EU) No 1169/2011 of the European Parliament and Council [26].

Each of the three samples in each group was analysed three times in the case of ADF, ADL, NDF and crude protein (*n* = 9). Three samples from each group were analysed twice in the case of crude fat and ash content (*n* = 6). Each MTOC flour fraction was analysed three times (*n* = 3 for all analyses).

#### 2.3.2. Sample Extraction for Silymarin Analysis and Antioxidant Activity Determination

Prior to the extraction of silymarin complex, the dough and bread samples were milled into a fine powder. The MTOC flour fraction samples were lyophilised and directly used for the extraction. Approximately 0.2 g of the lyophilised sample was mixed with 10 mL of 80% methanol (*v/v*) in a 15 mL plastic centrifuge tube. The sample was then extracted in an ultrasound bath (at 40 kHz; ArgoLab, Verkon, Praha, Czech Republic) for two hours, and the tubes were gently shaken every 15 min. Then, the extract was centrifuged at 5869× *g* (5000 rpm; Hettich, Rotina 380 R, Tuttlingen, Germany) for 30 min (20 °C) and stored at −18 °C until analysis.

#### 2.3.3. HPLC-MS/MS Analysis of Silymarin Complex

Analysis of the silymarin complex, namely taxifolin, silychristin, silydianin, silybin A, silybin B, isosilybin A and isosilybin B, content in the dough, bread and MTOC flour fraction samples was carried out using a high-performance liquid chromatograph (Dionex UltiMate 3000, Dionex, Sunnyvale, CA, USA) coupled to a triple quadrupole tandem mass spectrometer equipped with an electrospray ionization source (Agilent 6420 QqQ, Agilent Technologies, Santa Clara, CA, USA). Five µL of the extracted and appropriately-diluted sample was injected into the system. The separation was accomplished using a Phenomenex Kinetex C18 core-shell column (2.6 µm, 150 × 2.1 mm) at 35 °C. Two mobile phases were used: A, composed of 5% methanol with 0.5% formic acid (*v*/*v*) and B, composed of 100% methanol. The mobile phase flow rate was set at 0.2 mL/min and the elution gradient was set as follows: 0–15 min: 33% B; 15–20 min: 33–38% B; 20–35 min: 38% B; 35–40 min: 38–33% B. Re-equilibration of the column took five minutes before the injection of the next sample.

After the separation, the samples were introduced into the mass spectrometer operating in negative ionisation mode with the following settings: the capillary voltage was set at −3.5 kV, the flow rate of drying gas (N_2_) was 11 L/min with a pressure of 35 psi and temperature of 350 °C. The multiple reaction monitoring (MRM) mode was used for the quantification of the target compounds. The optimised parameters of the MRM mode for each compound are presented in Supplementary Material Table S2 (transition ions, fragmentor, collision energy and cell accelerator voltages), together with a six-point calibration curve linear range and the calibration curve correlation coefficients.

Results are expressed as mg/g DM (dry matter). Three measurements were conducted per group of dough, bread, or flour (*n* = 3).

#### 2.3.4. Antioxidant Activity

Two spectrophotometric measurement methods were selected to express the antioxidant activity of the samples according to Bárta et al. [8]: the DPPH (2,2-diphenyl-1-picrylhydrazyl) method and the ABTS (2,2′-azino-bis(3-ethylbenzothiazoline-6-sulfonic acid) method.

DPPH method: The sample extract (25 µL) was mixed with 975 µL of methanolic DPPH solution adjusted to an absorbance of 0.8 at 515 nm. The reaction mixture was kept in the dark at room temperature for two hours. Afterwards, the absorbance was measured at 515 nm against methanol using a Thermo Scientific Biomate 5 spectrophotometer (Thermo Fisher Scientific, Waltham, MA, USA). DPPH solution without the sample served as the blank. Trolox was used to prepare a calibration curve and results are expressed as mg of Trolox equivalent per gram of sample on a DM basis (mg TE/g DM). Three samples per group were analysed, thus *n* = 3.

ABTS method: Firstly, ABTS (54.9 mg) reagent together with 1.0 g MnO_2_ was dissolved in 20 mL of 5 mM phosphate buffer (pH 7.0) to produce ABTS^•+^ radicals within 30 min. The solution containing the radicals was then diluted with 5 mM phosphate buffer and the absorbance at 734 nm was adjusted to 0.8. Then, 0.1 mL of the sample extract was added to 1 mL of the working ABTS^•+^ radical solution. This mixture was then left to react for 1 min in the dark. Finally, the absorbance at 734 nm was measured using the same spectrophotometer as that used in the DPPH method. The results are expressed as mg TE/g DM. Three samples per group were analysed (*n* = 3).

### 2.4. Physical Analyses

#### 2.4.1. Specific Volume of the Bread Loaves

The baked bread loaves were left to cool to room temperature, weighed and then their volume was measured by the rapeseed displacement method using an OBK volumeter (Mezos, Náchod, Czech Republic) [27]. The specific loaf volume was expressed as cm^3^/g and was calculated as loaf volume divided by its weight. Three breads per group were analysed (*n* = 3).

#### 2.4.2. Colour Analysis of the Breads

The colour analysis of the bread crumb was accomplished using a ColorEye XTH spectrophotometer colour analyser (Gretag Macbeth, New Windsor, NY, USA) based on the modified method of Bedrníček et al. [28]. The results were presented in the CIE L*a*b* colour system. Three breads per group were analysed and each bread was analysed twice (*n* = 6).

#### 2.4.3. Texture Analysis of the Bread Loaves and Storage Experiment

The bread samples were put into a high-density polyethylene bag and stored for 96 h at room temperature in the dark to simulate the usual domestic storage conditions. During the storage, the hardness of the bread samples was analysed after the cooling of the bread (0 h, fresh bread), and then after 24, 48, 72 and 96 h.

The texture hardness of the bread crumb was measured by a TA.XT Plus texturometer (Stable Micro Systems, Surrey, UK) according to Moore et al. [29]. The analyser, equipped with a 50 kg load cell and cylindrical probe (diameter of 50 mm), had the following settings: pre-test speed of 10 mm/s, test speed of 2 mm/s and post-test speed of 5 mm/s with a trigger force of 20 g. Slices of 2.5 cm thickness were compressed to 60% of their original height and the force peak was taken as the hardness of texture. Three breads per group were analysed (*n* = 3) and the results are expressed as N (newton).

### 2.5. Sensory Analysis

The sensory properties of the breads were evaluated by a 14-member semi-trained sensory panel based on the modified methodology described by Bedrníček et al. [30]. Bread slices (randomly marked with a three-digit number) were served on white plates simultaneously to let the panellists directly compare the samples. The panellists were asked to evaluate the pleasantness of colour, odour, texture and taste, and the degree of bitterness, as well as the overall impression. These attributes were evaluated using a 10 cm unstructured hedonic or intensity scale (0 = dislike extremely, 10 = like extremely, for colour, odour, texture, taste and overall impression; and 0 = not present, 10 = most intense, for bitter taste intensity). The panellists then put a mark on the scale to characterize each of the attributes. The values obtained from all panellists were averaged, hence *n* = 14. Water was served as a taste neutraliser and was drank before the judging of the next sample.

### 2.6. Statistical Analysis

The results of all analyses are presented as mean ± standard deviation. Statistical analysis of obtained data was conducted using Statistica 12 software (StatSoft, Tulsa, OK, USA). To evaluate the statistically-significant effect of flour fraction additions into breads on basic chemical composition, sensory analysis, colour, texture and specific loaf volume analysis, one-way analysis of variance (ANOVA) was used. The same statistical test was used to detect significant differences between flour-fraction samples in all conducted analyses. Two-way analysis of variance was used to determine statistically significant differences between samples (doughs and breads) fortified with the different flour fractions (factor one) and between each processing step (before rising, after rising and after baking; factor two) in the case of silymarin analysis and antioxidant activity. Two-way ANOVA was also used for the data analysis in the case of the storage experiment, where the first factor was different flour-fraction addition and the second factor was storage time. Fisher’s LSD post hoc test was then applied to find out the differences between groups. All statistical tests were conducted at a significance level of α = 0.05; therefore, if *p* < 0.05, differences were considered as statistically significant.

## 3. Results and Discussion

### 3.1. Chemical Analyses

#### 3.1.1. Nutritional Characteristics of Breads with Milk Thistle Oilseed Cake Flour Fractions

The chemical composition or nutritional characteristics of foods are a very important aspect in terms of a healthy diet. The basic chemical composition (including crude protein, crude fat, ash, fibre, NFE, water content and energy) of the fortified GF breads is presented in Table 2.

GF bread often contains lower amounts of protein than gluten-containing bread [5]. In this study, the crude protein content in the control bread was 5.06%, however, with the addition of MTOC flour fractions the content significantly (*p* < 0.05) increased up to 8.07% in the case of the fine flour fraction. The lowest increase in crude protein was observed in the bread with the coarse fraction (5.59%). In general, with decreasing particle size of the flour fraction, the crude protein content increased. From this point of view, it seems advisable to use MTOC flour fractions, especially finer fractions, as a source of proteins for GF bread.

Regarding fat content, a similar trend was observed, where the lowest content was measured also in the control bread (4.56%) and the highest in bread containing fine flour fraction, 8.07%, which is an almost two-fold increase compared to the control. The crude fat increased in the enriched breads due to the presence of residual fat in the flour fractions, where crude fat ranged between 9.40 and 16.66%, as shown in Table 1. Higher content of fat can enhance nutritional value, and it was reported by Fathi-Achachlouei and Azadmard-Damirchi [12] that milk thistle oil contains 70–80% unsaturated fatty acids; specifically 50% linoleic acid and 20–30% oleic acid.

Incorporation of the MTOC flour fractions into GF bread led to significantly (*p* < 0.05) higher levels of insoluble dietary fibre for all fractions (cellulose, hemicellulose and lignin). The highest levels of cellulose, hemicellulose and lignin were found in the breads with coarse and medium fractions, while a slightly lower content was found in breads with the unsieved fraction. Bread samples with the fine fraction had the lowest content of fibre among all the fortified breads, although it was still significantly (*p* < 0.05) more than that in the control bread. Thus, it seems that MTOC, especially the coarse fraction, is a good source of fibre. This finding is in agreement with Bortlíková et al. [31], who also reported that biscuits with milk thistle flour increased total dietary fibre content compared to a control. In addition, the authors remark that milk thistle flour fibre mostly consists of insoluble dietary fibre. From this perspective, it is suitable to use to increase the fibre content in GF breads, since a GF diet is often characterized by a reduced intake of dietary fibre [32].

The content of ash in the breads with MTOC flour fractions was found to be in this order: fine > unsieved > coarse ~ medium > control, which again shows that the content increased significantly (*p* < 0.05) after fortification with MTOC flour fractions and the order is opposite to that of crude protein and fibre content. As in the case of fibre, Matos Segura and Rosell [32] also point out that a GF diet is often poor in minerals. However, there are no reports in the literature regarding the exact mineral composition of milk thistle achenes.

NFE is a calculated parameter and is what remains after the subtraction of the sum of crude protein, crude fat, total insoluble fibre, ash and water. Therefore, it consists of components that were not determined by direct chemical methods in this study e.g., starch, sugars and soluble dietary fibre. The effect of incorporation of the MTOC flour fractions into GF bread was significant (*p* < 0.05), more specifically, the content of NFE decreased from 48.43% (control bread) to 40.47% (bread with the fine fraction), whereas the breads with unsieved, coarse and medium flour fractions had 42.22, 41.97 and 41.68% NFE, respectively. However, the fortified samples did not differ from each other (*p* > 0.05). The recipe of the control bread was based mainly on starchy ingredients (Supplementary Materials Table S1) that contain only a very low amount of fibre. Thus, we assume that most of the NFE is composed of starch. In fact, Bortlíková et al. [31] reported that milk thistle flour is almost starch-free; thus, the addition of milk thistle flour fractions can reduce the excessive amount of starch in GF bakery products. According to Melini and Melini [3], GF diets formulated, for example, with corn flour, usually have a higher amount of starch, which results in an increased glycaemic index; and if such food is consumed over a longer time, it may increase the risk of developing metabolic syndromes in coeliac people, as was shown in several epidemiological studies.

The energy value of food is closely related to its chemical composition. The fortified bread samples showed statistically lower (*p* < 0.05) energy content (1058–1084 kJ/100 g) compared to the control samples (1122 kJ/100 g). Although it was reported by Cornicelli et al. [5] that GF bread usually has lower energy than gluten-containing bread, it should be also taken into account that energy intake in a GF diet is mainly caused by the higher content of carbohydrates [1]. With respect to enriched breads, more energy is represented by fat and less by NFE, which is, as mentioned above, represented mainly by starch. Thus, the energy profile is more suitable for rational nutrition if we consider the recommendation of Trumbo et al. [33] that 10–35% of energy should be covered by proteins, 20–35% by fat and 45–65% by carbohydrates. The detailed energy profile of the breads is shown in Supplementary Materials Table S3.

The water content in all the breads was very similar (36.31–37.02%) and did not differ significantly (*p* > 0.05).

In general, the addition of the MTOC flour fractions had a significant effect (*p* < 0.05) on the basic chemical composition of the bread samples, except for water content. The changes in bread chemical composition after the addition of the MTOC flour fractions reflect the chemical composition of the flour fractions themselves, as shown in Table 1. The sieving to the finest flour fraction increases the content of protein, fat and ash and decreases the content of insoluble fibre fractions and NFE. Oghbaei and Prakash [14] generally explained this phenomenon as due to the nature of fibre, which is hard to pulverize and thus cannot pass through a sieve, and the nature of endosperm (or similar tissue containing protein, starch or fat), which is easily milled, therefore passing through sieves. As a result of this, fibre is often contained in coarser fractions, while protein, starch (if present), fat and ash are usually found in finer fractions. This indicates the distribution of individual nutrients or chemical components in milk thistle achenes, meaning that the centre (kernel) of the achene is rich in protein, fat and ash, but poor in fibre content. This phenomenon was also documented by Bárta et al. [8] who prepared flour fractions differing in particle size from oilseed cakes from different oilseed crops (flax, hemp, milk thistle, poppy, pumpkin, rapeseed, safflower and sunflower). In most cases, sieving to finer fractions increased the content of protein, fat and ash and decreased the content of carbohydrates.

#### 3.1.2. HPLC-MS/MS Analysis of Silymarin and Antioxidant Activity in the Doughs and Breads

Seven main constituents of silymarin complex were monitored in the doughs and breads using HPLC-MS/MS, namely taxifolin, silychristin, silydianin, silybin A, silybin B, isosilybin A and isosilybin B. The concentration of these compounds in the dough and bread samples is presented in Table 3. In addition, the silymarin content in the unsieved, coarse, medium and fine MTOC flour fractions of the Mirel variety is shown in Table 1. Unsurprisingly, silymarin was not detected in the control group. Conversely, all seven compounds were found in all samples containing MTOC flour fractions.

The dominant compound in all samples was silybin B, followed by silybin A, silychristin, isosilybin A, isosilybin B and silydianin, while the least abundant was taxifolin. These proportions correspond with the pure flour fractions of the Mirel variety.

Regarding the effect of the addition of different MTOC flour fractions on the content of silymarin in the dough and bread samples, it is evident that the coarse and medium fractions are very similar and are the richest source of silymarin. A slightly lower content was found in the samples fortified with the unsieved fraction and a very low concentration of silymarin was detected in the samples containing the fine fraction. Again, this trend is in accordance with the content of silymarin in the pure flour fractions. Our hypothesis was that the content would be highest in samples containing the coarse fraction because we expected that silymarin accumulates mainly in the epidermis or outer organs of the achenes since many plants synthesize and store polyphenols near the surface of the seed or fruit as a defence against UV light or other organisms e.g., microorganism and herbivores [34]. However, this was not confirmed and the highest silymarin content was found in the samples with the medium fraction and also in the pure medium flour fraction. As suggested by AbouZid et al. [11], silymarin is mainly contained in the pericarp; but, in its outmost layer (epidermis), it is not present. We can assume that this epidermis layer is mostly present in the coarse flour fraction and as a result, samples with the medium fraction have the highest content of flavonolignans. Moreover, these authors also reported that the kernel of the achene contains almost no silymarin, which again corresponds with the fact that the samples with the finest fraction had the lowest content of silymarin.

Food processing methods can modify both the structure and quantity of polyphenols in foods, which can result in altered properties [22]. Therefore, another factor that was studied was whether the silymarin content in the doughs and breads enriched with MTOC flour fractions could be affected by the rising of the dough or baking of the bread. Thus, the dough samples were measured before and after rising and after baking. Table 3 shows the results. Generally, rising and baking significantly (*p* < 0.05) affected the content of silymarin and its individual constituents.

The rising of the dough at 33 °C for 60 min had a positive effect on all flavonolignans because the content in the doughs grew significantly (*p* < 0.05) after rising. In particular, the increase was 13% on average, but the highest increase was observed in the case of taxifolin (17%) and the lowest in the case of silydianin and isosilybin B (8% for both). It was reported that bioprocessing, such as fermentation, including dough rising, could release some of the polyphenolic compounds [22,35]. The reason for this could be that polyphenols can come into contact with some fractions of dietary fibre or starch and bind to them, which makes them largely inaccessible [36]. However, as proposed by Arfaoui [21], fermenting microorganisms (e.g., those causing dough rising) could release polyphenols due to their enzymatic activity. It must be mentioned that the added *S. cerevisiae* (as a part of the bread recipe in this study) are not the only microorganisms involved in dough rising, since many other microorganisms are naturally present in flour and become active during rising. In addition to microbial enzymatic activity, it is also possible that enzymes of plant origin present naturally in flours can be activated [37] and help with the release of polyphenols. Afterwards, the extraction from the food matrix could be easier compared to the non-fermented matrix and this may also be aligned with better bioavailability. It remains unknown whether silymarin flavonolignans are present in free form or are somehow held by some kind of bond in milk thistle achenes and consequently in the flour fractions.

The next step in the breadmaking was baking at 170 °C for 45 min. The internal temperature of the dough/bread was monitored using a puncture thermometer. In the first phase of baking, the internal temperature rose from 33 °C (rising temperature) during the first 15 min and then, after stabilization, it ranged between 95 and 100 °C for the remaining 30 min until the baking was done. Baking under these specific conditions affected all silymarin flavonolignans significantly (*p* < 0.05). Approximately a 20% decrease was observed for the whole silymarin complex, thus 80% remained after baking. Individual flavonolignans, except silydianin, decreased by about 25–17%. An interesting finding was that silydianin exhibited the highest loss during baking and only 52% remained. The results imply that the compounds are moderately thermally stable, but silydianin is sensitive to elevated temperatures. This finding might be related to the different structure of silydianin compared to other flavonolignans, which is unique and somewhat complicated due to the presence of a keto group next to the hemiacetal moiety [38]. To the best of our knowledge, this study is the first one dealing with the thermal stability of silymarin in a real food matrix under real food processing conditions. There are a few papers about the thermal stability of silymarin flavonolignans; however, these are rather aimed at laboratory experiments. For example, Korany, et al. [39] exposed pure working standards of flavonolignans to dry heat (105 °C, 4 h) and neutral hydrolysis in water (70 °C, 4 h) and reported that under these conditions silymarin flavonolignans are stable. Duan et al. [40] studied the subcritical water extraction parameters of silymarin from milk thistle achenes. They found that 20% of silymarin started degrading after 20 min of being exposed to extraction at 100 °C, which roughly corresponds with our results. Conversely, these authors did not observe a higher rate of silydianin degradation compared to other flavonolignans. From this, we can assume that the compounds in our samples degraded as a consequence of the presence of water and high temperatures, which resulted in their partial hydrolysis, or else degraded due to the combination of the aforementioned factors and interaction with other compounds.

Despite the changes in silymarin content during bread making, considerable amounts remained in finished breads. Thus, if we consider the consumption of a 200 g portion of bread per day, then a person would ingest 436, 504, 480, or 126 mg of silymarin in the case of bread with unsieved, coarse, medium, or fine flour fraction, respectively. As reported by Gillessen and Schmidt [19], the recommended daily dosage (depending on the commercial formulation) is between 420 and 600 mg, whereas 120 mg three times a day was the usual amount used in most of the clinical trials focusing on the influence of silymarin on the liver. Thus, the breads with 10% unsieved, coarse and medium flour fraction made in this study, if consumed in the mentioned amount, provide the recommended amount of silymarin intake per day. Another question is, however, the bioavailability of silymarin, which is lipophilic in nature [19], if it was ingested in the form of bread; because the presence of different macronutrients (e.g., proteins, polysaccharides and lipids) can change its solubility and bioavailability.

Although there are a great number of natural products that are currently used as antioxidant substrates, the search for new agents with antioxidant activity remains a burgeoning interest [41]. Silymarin flavonolignans possess effective in vitro antioxidant properties [20]; hence antioxidant activity determined by two assays (DPPH and ABTS) was measured in all dough and bread samples (Table 4) in all processing phases, in addition to the HPLC-MS/MS silymarin analysis.

Antioxidant activity mirrored the trend in silymarin content in the dough and bread samples. Thus, a very significant correlation was observed for the DPPH assay (r = 0.925, *p* < 0.05) and the ABTS assay (r = 0.996, *p* < 0.05). This means that the samples containing silymarin flour fractions had significantly (*p* < 0.05) higher antioxidant activity than the control samples. Also, the samples with the medium fraction exerted the highest antioxidant activity, followed by the samples with the coarse fraction, then the unsieved fraction, with the lowest antioxidant activity exhibited by the samples with the fine fraction.

In terms of the influence of the processing phase on the antioxidant activity of the samples, we obtained relatively interesting results. Although a high correlation with silymarin content was found, as mentioned above, the DPPH assay did not show any significant (*p* > 0.05) increase in antioxidant activity in the fortified dough samples after rising, since only a 4% increase on average was observed. Similarly, baking also did not affect antioxidant activity significantly (*p* > 0.05) using the DPPH method, because only an 8% loss (on average) was observed with relatively high variability (± 9%) among samples. On the other hand, the ABTS assay showed more precise results which also corresponds with a higher correlation coefficient with silymarin content almost reaching 1 (as also mentioned above). Moreover, both factors, rising and baking, had a significant effect (*p* < 0.05) on antioxidant activity. Thus, a significant increase (5%) was observed after rising and a 9% loss after baking. A probable explanation for the discrepancy between the DPPH and ABTS results may be found in the different specificity and sensitivity of these assays. It was reported that ABTS antioxidant capacity is usually higher compared to that measured by DPPH assay [42]. This could be due to the greater sensitivity of the ABTS method, such that it could distinguish differences between our samples.

Even though baking of the bread samples resulted in approximately a 20% loss of silymarin flavonolignans, it did not affect the antioxidant activity to such an extent, as 92 and 91% of antioxidant activity remained for DPPH and ABTS, respectively. Polovka and Suhaj [43] explained the mechanism by which antioxidant activity decreases only slightly in thermally-treated foods. The authors state that natural antioxidants in food can decompose during thermal processing; however, the heat generates new antioxidant content, such as Maillard reaction products, that equalizes the loss of antioxidant activity.

### 3.2. Qualitative Properties of the Bread Samples

**Figure 1:**
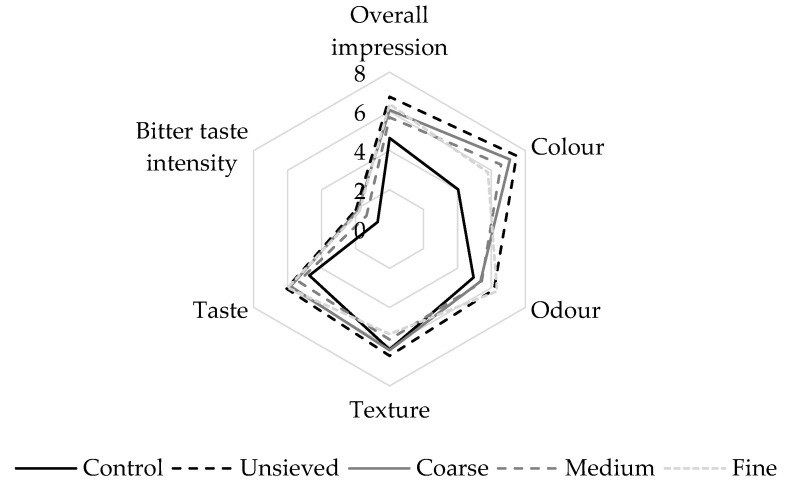
Sensory analysis of the control bread and breads fortified with different fractions of milk thistle oilseed cake.

GF breads usually have worse qualitative parameters (e.g., texture, sensory properties) compared to gluten-containing breads, which has compelled scientists to look for new ingredients or develop new recipes that enhance the quality of GF breads [44]. Thus, we examined the loaf-specific volume, colour, texture hardness (Table 5) and sensory properties (pleasantness of colour, odour, texture and taste, bitterness and overall impression; Figure 1) of the breads.

Specific loaf volume was significantly (*p* < 0.05) affected by the addition of the MTOC flour fractions; in particular, the volume decreased. The highest specific loaf volume was observed in the control group (2.76 cm^3^/g) and the lowest in the unsieved and coarse groups (both having 2.59 cm^3^/g). However, the specific loaf volume of the samples with the fine flour fraction did not differ significantly (*p* > 0.05) from that of the control group (2.69 cm^3^/g). This trend could probably be caused by a higher content of insoluble fibre, which could make the specific loaf volume lower, as summarized by Sabanis et al. [45]. On the other hand, proteins from alternative sources could partially replace the function of gluten [46]. Similar results were obtained by Bojňanská et al. [47], who also reported a decrease in specific loaf volume after the addition of 5% of defatted milk thistle seed flour into wheat bread. The defatted flour could correspond with the unsieved fraction used in this study. It should be highlighted that a lower specific volume does not necessarily mean worse bread quality.

The colour of the bread was substantially affected by the addition of the MTOC flour fractions. Thus, all three colour parameters (L*, a* and b*) were affected significantly (*p* < 0.05). The control bread was very light, while the fortified breads were much darker and had an appearance similar to rye bread [48], as illustrated in Supplementary Materials Figure S1. Heiniö et al. [48] remarked that a darker colour can be more attractive for some consumers because it evokes a product containing, e.g., more fibre, making it healthier.

Bread staling is a process deteriorating the quality of a bread that is stored for a longer period and is characterised mainly by a harder texture. In order to assess bread staling during storage at room temperature, the texture of bread samples packed in a high-density polyethylene bag was measured 0 (fresh), 24, 48, 72 and 96 h after baking. The design of the storage experiment was chosen to simulate the usual conditions at home or a supermarket. The texture hardness of the breads was affected significantly (*p* < 0.05) by both factors (MTOC flour fraction and storage time). Samples containing MTOC flour fractions were much harder than the control bread, which can be linked to the fact that they contained more insoluble fibre, proteins and fat, which usually affect texture [49]. The hardness increased in the order: control < medium < coarse ~ unsieved < fine.

Unsurprisingly, the hardness increased over time in all samples; but an interesting fact was that the increment of hardness in all samples was not the same. The texture of the samples with MTOC flour fractions did not harden during storage to such an extent as the control bread. For example, the control samples had an initial hardness of 80.9 N, which grew to 233.4 N after 96 h, almost three times higher. In contrast, in the breads with the MTOC fine fraction, the increment in hardness was only one-third.

In this particular recipe, adding MTOC flour fractions to GF breads somehow slowed down staling of the bread compared to the control group, even though the samples containing the MTOC flour fractions were harder initially than the control.

A fourteen-member panel evaluated the following sensory characteristics: pleasantness of colour, odour, texture and taste, bitterness and overall impression (Figure 1). The panel found differences among all samples in all sensory traits; however, they were statistically significant (*p* < 0.05) only in the case of the pleasantness of colour and overall impression. Therefore, the differences within the remaining characteristics were insignificant (*p* > 0.05). Regarding the colour, the control samples received the worst score (4.0), followed by the bread with the fine flour fraction (5.8). On the other hand, the best evaluation was observed in the samples with the unsieved fraction (7.5). This result could be due to the fact that darker breads could be more positively accepted due to the expectation of higher health benefits [48], as already mentioned. Although most of the assessed sensory traits were not influenced to a great extent, the bread with the unsieved fraction had the highest score of the overall impression. Thus, in terms of sensory acceptance, the unsieved fraction would be more accepted by consumers if these breads were produced on an industrial scale. Nevertheless, it must be added that sensory properties are also strongly dependent on specific recipes and baking technology, and in different recipes, the differences wouldn’t be so considerable.

## 4. Conclusions

This paper brings novel findings and describes the possible utilization of milk thistle oilseed cake as an ingredient for GF bread for the first time. Based on the results shown in this study, it seems that milk thistle oilseed cakes are a promising ingredient for GF bakery products. If the oilseed cakes are sieved and separated into different fractions and then added into GF bread, the resulting enriched breads will have different chemical compositions and properties. All enriched breads had a higher content of protein, fat, fibre and silymarin, but, a lower content of NFE, compared to the control GF bread. Differences between the fortified breads were also obvious. The breads with the coarse fraction had a higher content of fibre and a lower content of protein, fat and ash compared to breads with the fine fraction, which showed an opposite trend. Moreover, during bread making, we observed a certain increase in silymarin content after dough rising, but a moderate decrease in silymarin after baking, indicating sensitivity to heat. It should be mentioned that this might also be related to the presence of water and other compounds that can react with silymarin. If rising and baking are compared, then baking has a more significant impact on silymarin quantity and quality, since the most sensitive compound was silydianin. In terms of the sensory properties of the breads, the addition of the fractions caused a much darker colour of the crumb and also a harder texture compared to the control. At the same time, the enriched breads were preferred more than the control bread by the assessor panel.

Practical and economic aspects should also be taken into account. Every processing step, including sieving/fractionation, is time- and cost-consuming and producing more than three fractions might be unnecessary. We, therefore, suggest that it might be enough to produce a very-fine fraction, e.g., with a particle size of <200 µm, that can be considered a rich source of proteins and a coarser fraction (>200 µm) with higher content of fibre and silymarin, which also increases the yield of both fractions. The two fractions might have different applications in the food industry. The finer fraction might enhance the nutrition profile of GF bread recipes that are mainly based on starchy ingredients, or can be used, for example, to produce “high-protein” products. On the other hand, the coarser fraction might be used for breads as a source of fibre-rich material that also delivers high amounts of silymarin to the final product. However, we wouldn’t recommend an addition level higher than 10% for any fraction.

After considering all the results, a few questions arise from the present study that should be examined in the future, e.g., firstly, what is the bioavailability of silymarin from the GF breads, and secondly, can the fortified bread enhance liver function, or possess other health benefits related to silymarin?

## Figures and Tables

**Table 1:** Basic chemical composition, content of silymarin constituents and antioxidant activity in the milk thistle oilseed cake flour fractions.

Parameter	Milk Thistle Oilseed Cake Flour Fraction			
	Unsieved	Coarse	Medium	Fine
Basic chemical composition (g/100 g FW)				
Crude protein	19.26 ± 1.21 ^B^	19.16 ± 0.57 ^B^	18.86 ± 0.23 ^B^	28.25 ± 0.53 ^A^
Fat	11.23 ± 0.19 ^B^	9.40 ± 0.05 ^D^	10.39 ± 0.04 ^C^	16.66 ± 0.12 ^A^
Cellulose	23.36 ± 0.26 ^B^	25.83 ± 0.38 ^A^	26.40 ± 0.29 ^A^	11.92 ± 0.50 ^C^
Hemicellulose	2.74 ± 0.24 ^C^	5.32 ± 0.20 ^A^	3.14 ± 0.09 ^B^	5.22 ± 0.37 ^A^
Lignin	16.53 ± 0.18 ^C^	18.29 ± 0.38 ^A^	17.26 ± 0.29 ^B^	13.27 ± 0.35 ^D^
Total insoluble fibre	42.63 ± 0.22 ^C^	49.44 ± 0.07 ^A^	46.81 ± 0.02 ^B^	30.41 ± 0.19 ^D^
Ash	6.23 ± 0.03 ^B^	5.52 ± 0.01 ^C^	5.43 ± 0.01 ^D^	8.78 ± 0.01 ^A^
Nitrogen-free extract *	16.88 ± 16.67 ^A^	11.89 ± 1.30 ^BC^	14.11 ± 0.74 ^B^	10.71 ± 1.79 ^C^
Water	4.50 ± 0.60	5.62 ± 0.82	5.32 ± 0.56	5.98 ± 1.01
Energy (kJ/100 g)	1365 ± 6 ^B^	1263 ± 16 ^D^	1312 ± 10 ^C^	1516 ± 16 ^A^
Silymarin constituents (mg/g DM)				
Taxifolin	0.66 ± 0.08 ^C^	0.88 ± 0.04 ^B^	1.11 ± 0.02 ^A^	0.26 ± 0.00 ^D^
Silychristin	7.72 ± 0.51 ^C^	10.49 ± 0.56 ^B^	11.87 ± 0.25 ^A^	3.26 ± 0.01 ^D^
Silydianin	0.92 ± 0.17 ^C^	1.33 ± 0.07 ^B^	1.85 ± 0.17 ^A^	0.35 ± 0.03 ^D^
Silybin A	7.91 ± 0.60 ^B^	10.56 ± 0.60 ^A^	11.73 ± 0.47 ^A^	3.33 ± 0.04 ^C^
Silybin B	13.07 ± 0.98 ^B^	17.28 ± 1.24 ^A^	19.10 ± 0.98 ^A^	4.69 ± 0.06 ^C^
Isosilybin A	3.27 ± 0.22 ^B^	4.24 ± 0.32 ^A^	4.72 ± 0.23 ^A^	1.30 ± 0.02 ^C^
Isosilybin B	1.05 ± 0.08 ^B^	1.38 ± 0.10 ^A^	1.55 ± 0.06 ^A^	0.43 ± 0.01 ^C^
Sum of flavonolignans	34.60 ± 2.64 ^B^	46.16 ± 2.92 ^A^	51.93 ± 1.85 ^A^	13.62 ± 0.07 ^C^
Antioxidant activity (mg TE/g DM)				
DPPH	22.39 ± 0.73 ^B^	23.06 ± 0.91 ^AB^	24.18 ± 0.68 ^A^	17.98 ± 2.32 ^C^
ABTS	58.25 ± 2.27 ^B^	67.56 ± 2.23 ^A^	68.77 ± 1.74 ^A^	27.37 ± 1.04 ^C^

Results are expressed as means ± standard deviation (*n* = 3); ^A–D^ Values with different superscripts within a row differ significantly (*p* < 0.05) based on Fisher’s LSD test; DPPH = 2,2-diphenyl-1-picrylhydrazil; ABTS = [2,2′-azinobis-(3-ethylbenzothiazoline-6-sulfonic acid)]; TE = trolox equivalent; DM = dry matter; FW = fresh weight; * Nitrogen-free extract was calculated as follows: 100 -(water, crude protein, fat, total insoluble fibre and ash content).

**Table 2:** Basic chemical composition of the control bread and the bread samples fortified with different flour fractions of milk thistle oilseed cake.

Chemical Composition (g/100 g FW)	Added Fraction				
	Control	Unsieved	Coarse	Medium	Fine
Crude protein	5.06 ± 0.26 ^E^	5.98 ± 0.43 ^C^	5.59 ± 0.32 ^D^	6.33 ± 0.32 ^B^	8.07 ± 0.22 ^A^
Crude fat	4.56 ± 0.35 ^D^	5.49 ± 0.12 ^B^	5.12 ± 0.15 ^C^	5.10 ± 0.11 ^C^	5.79 ± 0.03 ^A^
Cellulose	1.98 ± 0.046 ^C^	3.37 ± 0.15 ^A^	3.84 ± 0.27 ^A^	3.80 ± 0.13 ^A^	2.81 ± 0.07 ^B^
Hemicellulose	1.12 ± 0.93 ^B^	1.91 ± 0.33 ^A^	2.24 ± 0.06 ^A^	2.29 ± 0.05 ^A^	1.98 ± 0.02 ^A^
Lignin	0.16 ± 0.09 ^C^	1.34 ± 0.16 ^A^	1.43 ± 0.06 ^A^	1.44 ± 0.05 ^A^	0.77 ± 0.07 ^B^
Total insoluble fibre *	3.26 ± 0.99 ^C^	6.62 ± 0.62 ^A^	7.51 ± 0.27 ^A^	7.53 ± 0.09 ^A^	5.56 ± 0.67 ^B^
Ash	2.39 ± 0.01 ^C^	2.99 ± 0.01 ^AB^	2.87 ± 0.15 ^B^	2.87 ± 0.01 ^B^	3.09 ± 0.02 ^A^
Nitrogen-free extract **	48.43 ± 1.68 ^A^	42.22 ± 0.27 ^B^	41.97 ± 0.26 ^B^	41.68 ± 0.68 ^B^	40.47 ± 1.85 ^B^
Water	36.31 ± 1.34	36.7 ± 0.89	36.93 ± 0.49	36.49 ± 0.99	37.02 ± 1.39
Energy (kJ/100 g)	1122 ± 12 ^A^	1075 ± 8 ^B^	1058 ± 8 ^B^	1065 ± 18 ^B^	1084 ± 29 ^B^

Results are expressed as mean ± standard deviation (*n* = 3); ^A– E^ Values with different superscripts within a row differ significantly (*p* < 0.05) based on Fisher’s LSD test; * total insoluble fibre was calculated as the sum of cellulose, hemicellulose and lignin; ** calculated; FW = fresh weight.

**Table 3:** Content of silymarin constituents in dough samples before and after raising and bread samples fortified with different milk thistle oilseed cake flour fractions.

Added Fraction	Processing Phase	Concentration of Silymarin Constituents (mg/g DM)							
		Taxifolin	Silychristin	Silydianin	Silybin A	Silybin B	Isosilybin A	Isosilybin B	Total Concentration
Control	Dough BR	ND ^f^	ND ^i^	ND ^d^	ND ^h^	ND ^h^	ND ^h^	ND ^g^	-^h^
	Dough AR	ND ^f^	ND ^i^	ND ^d^	ND ^h^	ND ^h^	ND ^h^	ND ^g^	-^h^
	Bread	ND ^f^	ND ^i^	ND ^d^	ND ^h^	ND ^h^	ND ^h^	ND ^g^	-^h^
Unsieved	Dough BR	0.08 ± 0.01 ^cd^	0.91 ± 0.05 ^d^	0.13 ± 0.03 ^a^	0.91 ± 0.06 ^d^	1.50 ± 0.07 ^cd^	0.37 ± 0.01 ^c^	0.13 ± 0.01 ^c^	4.02 ± 0.23 ^c^
	Dough AR	0.10 ± 0.00 ^b^	1.00 ± 0.01 ^c^	0.15 ± 0.01 ^a^	0.98 ± 0.02 ^c^	1.62 ± 0.05 ^b^	0.41 ± 0.01 ^b^	0.13 ± 0.01 ^bc^	4.39 ± 0.07 ^b^
	Bread	0.07 ± 0.00 ^d^	0.77 ± 0.06 ^f^	0.08 ± 0.01 ^b^	0.80 ± 0.05 ^e^	1.34 ± 0.10 ^e^	0.30 ± 0.02 ^e^	0.10 ± 0.01 ^e^	3.45 ± 0.24 ^e^
Coarse	Dough BR	0.07 ± 0.01 ^d^	0.89 ± 0.04 ^de^	0.15 ± 0.06 ^a^	0.87 ± 0.02 ^d^	1.51 ± 0.04 ^cd^	0.37 ± 0.01 ^c^	0.13 ± 0.01 ^c^	3.99 ± 0.10 ^c^
	Dough AR	0.09 ± 0.00 ^c^	1.02 ± 0.05 ^bc^	0.15 ± 0.03 ^a^	0.99 ± 0.99 ^bc^	1.66 ± 0.08 ^b^	0.41 ± 0.02 ^b^	0.14 ± 0.00 ^b^	4.45 ± 0.18 ^b^
	Bread	0.08 ± 0.00 ^cd^	0.89 ± 0.01 ^de^	0.09 ± 0.01 ^b^	0.92 ± 0.00 ^d^	1.54 ± 0.01 ^c^	0.34 ± 0.00 ^d^	0.12 ± 0.00 ^c^	4.00 ± 0.01 ^c^
Medium	Dough BR	0.10 ± 0.01 ^b^	1.06 ± 0.02 ^b^	0.17 ± 0.02 ^a^	1.04 ± 0.02 ^ab^	1.77 ± 0.03 ^a^	0.44 ± 0.01 ^a^	0.15 ± 0.00 ^a^	4.72 ± 0.08 ^a^
	Dough AR	0.11 ± 0.00 ^a^	1.13 ± 0.02 ^a^	0.16 ± 0.03 ^a^	1.08 ± 0.03 ^a^	1.82 ± 0.04 ^a^	0.46 ± 0.01 ^a^	0.15 ± 0.00 ^a^	4.91 ± 0.09 ^a^
	Bread	0.08 ± 0.01 ^d^	0.84 ± 0.04 ^e^	0.09 ± 0.01 ^b^	0.88 ± 0.05 ^d^	1.43 ± 0.06 ^d^	0.34 ± 0.01 ^d^	0.11 ± 0.01 ^d^	3.78 ± 0.08 ^d^
Fine	Dough BR	0.02 ± 0.00 ^e^	0.25 ± 0.02 ^g^	0.03 ± 0.00 ^c^	0.26 ± 0.03 ^g^	0.40 ± 0.05 ^f^	0.11 ± 0.01 ^g^	0.03 ± 0.00 ^f^	1.10 ± 0.11 ^f^
	Dough AR	0.03 ± 0.00 ^e^	0.32 ± 0.01 ^h^	0.03 ± 0.00 ^c^	0.32 ± 0.01 ^f^	0.51 ± 0.01 ^g^	0.13 ± 0.00 ^f^	0.04 ± 0.00 ^f^	1.38 ± 0.03 ^g^
	Bread	0.02 ± 0.00 ^e^	0.22 ± 0.02 ^g^	0.01 ± 0.00 ^c^	0.25 ± 0.02 ^g^	0.38 ± 0.04 ^f^	0.09 ± 0.01 ^g^	0.03 ± 0.00 ^f^	1.00 ± 0.10 ^f^
Compound released during raising (%)		17 ± 4	15 ± 9	8 ± 13	13 ± 9	12 ± 11	12 ± 8	8 ± 6	13 ± 9
Remaining compound after baking (%)		80 ± 11 ^A^	77 ± 7 ^A^	52 ± 9 ^B^	83 ± 7 ^A^	82 ± 8 ^A^	75 ± 6 ^A^	81 ± 8 ^A^	80 ± 7 ^A^

Results are expressed as mean ± standard deviation (*n* = 3); ^a–i^ Values with different superscripts within a column differ significantly (*p* < 0.05) based on Fisher’s LSD test; ^A,B^ Values with different superscripts within a row differ significantly (*p* < 0.05) based on Fisher’s LSD test; BR = before raising, AR = after raising; ND = not detected; DM = dry matter.

**Table 4:** Antioxidant activity (DPPH and ABTS) in doughs before and after raising and breads fortified with different milk thistle oilseed cake flour fractions.

Added Fraction	Processing Phase	Antioxidant Activity (mg TE/g DM)	
		DPPH	ABTS
Control	Dough BR	0.61 ± 0.02 ^g^	1.24 ± 0.02 ^i^
	Dough AR	0.61 ± 0.01 ^g^	1.25 ± 0.03 ^i^
	Bread	0.58 ± 0.07 ^g^	1.19 ± 0.06 ^i^
Unsieved	Dough BR	1.87 ± 0.11 ^d^	6.90 ± 0.41 ^ef^
	Dough AR	1.99 ± 0.06 ^cd^	7.28 ± 0.19 ^cd^
	Bread	1.96 ± 0.08 ^cd^	6.74 ± 0.09 ^f^
Coarse	Dough BR	2.02 ± 0.05 ^cd^	7.31 ± 0.05 ^c^
	Dough AR	2.09 ± 0.08 ^bc^	7.89 ± 0.20 ^ab^
	Bread	2.04 ± 0.11 ^bc^	7.09 ± 0.09 ^de^
Medium	Dough BR	2.18 ± 0.05 ^ab^	7.84 ± 0.11 ^b^
	Dough AR	2.26 ± 0.13 ^a^	8.11 ± 0.09 ^a^
	Bread	2.10 ± 0.06 ^bc^	7.05 ± 0.10 ^de^
Fine	Dough BR	1.67 ± 0.18 ^e^	2.97 ± 0.03 ^gh^
	Dough AR	1.69 ± 0.14 ^e^	3.03 ± 0.02 ^g^
	Bread	1.32 ± 0.06 ^f^	2.83 ± 0.04 ^h^
Antioxidant activity increased after raising (%)		4 ± 2	5 ± 2
Remaining antioxidant activity after baking (%)		92 ± 9	91 ± 3

Results are expressed as mean ± standard deviation (*n* = 3); ^a–i^ Values with different superscripts within a column differ significantly (*p* < 0.05) based on Fisher’s LSD test; BR = before raising, AR = after raising; DM = dry matter; DPPH = 2,2-diphenyl-1-picrylhydrazil; ABTS = [2,2′-azinobis-(3-ethylbenzothiazoline-6-sulfonic acid)]; TE = trolox equivalent.

**Table 5:** Specific volume, crumb colour and texture analysis of the control bread and breads fortified with different milk thistle oilseed cake flour fractions.

Parameter	Added Fraction				
	Control	Unsieved	Coarse	Medium	Fine
Vs (cm^3^/g)	2.76 ± 0.04 ^A^	2.59 ± 0.10 ^B^	2.59 ± 0.02 ^B^	2.60 ± 0.10 ^B^	2.69 ± 0.00 ^AB^
Crumb colour in CIE L*a*b system					
L	54.56 ± 2.71 ^A^	42.26 ± 1.29 ^B^	43.71 ± 0.95 ^B^	44.39 ± 2.70 ^B^	42.43 ± 2.19 ^B^
a	0.24 ± 0.17 ^B^	3.95 ± 0.30 ^A^	3.94 ± 0.35 ^A^	3.67 ± 0.27 ^A^	3.76 ± 0.36 ^A^
b	18.04 ± 0.64 ^A^	17.05 ± 0.41 ^B^	17.65 ± 0.66 ^AB^	17.40 ± 1.27 ^AB^	15.80 ± 0.88 ^C^
Hardness (N) of texture during storage					
0 h (fresh)	80.9 ± 11 ^dC^	133.6 ± 22 ^dB^	132.6 ± 17 ^dB^	107.5 ± 29 ^cBC^	224.7 ± 35 ^cA^
24 h	140.6 ± 14 ^cB^	206.9 ± 21 ^cA^	233.4 ± 20 ^cA^	150.9 ± 31 ^bB^	235.5 ± 31 ^cA^
48 h	191.3 ± 20 ^bB^	261.9 ± 12 ^bA^	274.2 ± 23 ^bA^	205.3 ± 27 ^aB^	241.7 ± 23 ^bcAB^
72 h	245.1 ± 18 ^aB^	319.5 ± 11 ^aA^	325.5 ± 19 ^aA^	239.5 ± 34 ^aB^	284.6 ± 1 ^abAB^
96 h	233.4 ± 27 ^aC^	284.3 ± 8 ^abAB^	314.3 ± 5 ^aA^	246.8 ± 54 ^aBC^	316.0 ± 14 ^aA^

Results are expressed as mean ± standard deviation (*n* = 3 for specific volume and texture hardness and *n* = 6 for colour analysis); ^a–d^ Values with different superscripts within a column differ significantly (*p* < 0.05) based on Fisher’s LSD test; ^A–C^ Values with different superscripts within a row differ significantly (*p* < 0.05) based on Fisher’s LSD test; Vs = specific volume of bread loaf, L = lightness, a = redness, b = yellowness.

## Data Availability

The data presented in this study are available in the article.

## Supplementary Materials

The following supporting information can be downloaded at: https://www.mdpi.com/article/10.3390/antiox11102022/s1, Figure S1: Pictures of crumb and crust of the control gluten-free bread (A) and the breads enriched with unsieved (B), coarse (C), medium (D) and fine (E) milk thistle oilseed cake flour fractions; Table S1: Recipes of the control bread and breads fortified with different fractions of milk thistle oilseed cake flour; Table S2: Settings of multiple reaction monitoring acquisition mode of the mass spectrometer for determination of silymarin complex; Table S3: Energy profile (%) in the control bread and the breads with unsieved, coarse, medium and fine milk thistle oilseed cake flour.
