# Supplementary material for: Milk Thistle Oilseed Cake Flour Fractions: A Source of Silymarin and Macronutrients for Gluten-Free Bread
Source: Antioxidants (Basel). 2022 Oct 13;11(10):2022. doi: 10.3390/antiox11102022 (PMC9598143; doi:10.3390/antiox11102022)

Supplementary Material for article: Milk Thistle Oilseed Cake Flour Fractions: A Source of Silymarin and Macronutrients for Gluten-Free Bread

Jan Bedrníček[1*], František Lorenc[1], Markéta Jarošová[2], Veronika Bártová[2], Pavel Smetana[1], Jaromír Kadlec[1], Dana Jirotková[1], Jan Kyselka[3], Eva Petrášková[4], Marie Bjelková[5], Petr Konvalina[6], Trong Nghia Hoang[6] and Jan Bárta[2]

[1]Department of Food Biotechnologies and Agricultural Products' Quality, Faculty Agriculture and Technology, University of South Bohemia in České Budějovice, Studentská 1668, 370 05 České Budějovice, Czech Republic

[2]Department of Plant Production, Faculty Agriculture and Technology, University of South Bohemia in České Budějovice, Na Sádkách 1780, 370 05 České Budějovice, Czech Republic

[3]Department of Dairy, Fat and Cosmetics, University of Chemistry and Technology Prague, Technická 5, 166 28 Prague 6 – Dejvice, Czech Republic

[4]Department of Animal Husbandry Sciences, Faculty Agriculture and Technology, University of South Bohemia in České Budějovice, Studentská 1668, 370 05 České Budějovice, Czech Republic

[5]Department of Legumes and Technical Crops, Agritec Plant Research, Ltd. Zemědělská 2520, 787 01 Šumperk, Czech Republic

[6]Department of Agroecosystems, Faculty Agriculture and Technology, University of South Bohemia in České Budějovice, Branišovská 1645, 370 05 České Budějovice, Czech Republic

[*]Corresponding author's email address: bedrnicek@fzt.jcu.cz, tel.: + 420 389 032 612

Table S1. Recipe of control bread and breads fortified with different fractions of milk thistle oilseed cake flour.

Ingredient	Amount of ingredients (%)	
	Control bread	Breads with 10 % of milk thistle oilseed cake flour fractions*
Sorghum flour	23.20	20.88
Corn flour	11.60	10.44
Corn starch	5.80	5.22
Potato starch	5.80	5.22
Tapioca starch	5.80	5.22
Guar flour	0.52	0.47
Milk thistle oilseed cake flour fractions	0	5.27
Sunflower oil	2.32	2.32
Salt	0.81	0.81
Dried yeasts	0.58	0.58
Water	43.56	43.56

*10 % of gluten-free flour blend was replaced by different milk thistle oilseed cake flour fractions: unsieved, coarse, medium and fine fraction.

Table S2. Settings of multiple reaction monitoring acquisition mode of mass spectrometer for determination of silymarin complex.

Compound	Precursor ion (*m*/*z*)	Product Ion (*m*/*z*)	Fragmentor voltage (V)	Collision energy (eV)	Cell accelerator voltage (V)	LOD (µg/ml)	LOQ (µg/ml)	Calibration curve linear range (ng/ml)	Calibration curve correlation coefficient (R^2)
Taxifolin	303	285	135	8	4	0.0001	0.0004	7.8 – 125.0	0.999
Silychristin	481	325	135	20	4	0.0011	0.0036	31.3 – 1000.0	0.993
Silydianin	481	151	135	20	4	0.0005	0.0017	7.8 – 250.0	0.997
Silybin A	481	301	135	20	4	0.0006	0.0020	61.9 – 989.6	0.998
Silybin B	481	301	135	20	4	0.0010	0.0033	252.6 – 1010.4	0.999
Isosilybin A	481	125	135	30	4	0.0014	0.0047	20.5 – 657.1	0.998
Isosilybin B	481	125	135	30	4	0.0002	0.0008	10.7 – 342.9	0.999

LOD = limit of detection, LOQ = limit of quantification; LODs and LOQs were calculated according to this formula: LOD, LOQ =$x = \frac{k \times \sigma}{S}$, where k is 3 or 10 for LOD or LOQ, respectively, σ is the baseline noise of the blank solution chromatogram and S is the slope of the calibration curve.

Table S3. Energy profile (%) in control bread and breads with unsieved, coarse, medium and fine milk thistle oilseed cake flour.

**Total energy represented by specific nutrient (%)**	**Fraction added to bread**				
	**Control**	**Unsieved**	**Coarse**	**Medium**	**Fine**
Crude protein	7.8±0.4^{D}	9.5±0.3^{C}	9.0±0.3^{C}	10.1±0.2^{B}	12.7±0.2^{A}
Crude fat	15.3±1.0^{D}	18.9±0.6^{AB}	17.9±0.5^{BC}	17.7±0.1^{C}	19.8±0.4^{A}
Total insoluble fibre	2.4±0.8^{C}	4.9±0.4^{AB}	5.7±0.2^{A}	5.7±0.1^{A}	4.1±0.6^{B}
Nitrogen-free extract	74.6±0.5^{A}	66.7±0.3^{B}	67.4±0.1^{B}	66.5±0.3^{B}	63.4±1.2^{C}

Results are expressed as means ± standard deviation (n = 3); $^{A-D}$Values with different superscripts within a row differ significantly ($p < 0.05$) based on Fisher's LSD test.

Figure S1. Pictures of crumb and crust of control gluten-free bread (A) and breads enriched with unsieved (B), coarse (C), medium (D) and fine (E) milk thistle oilseed cake flour fractions.

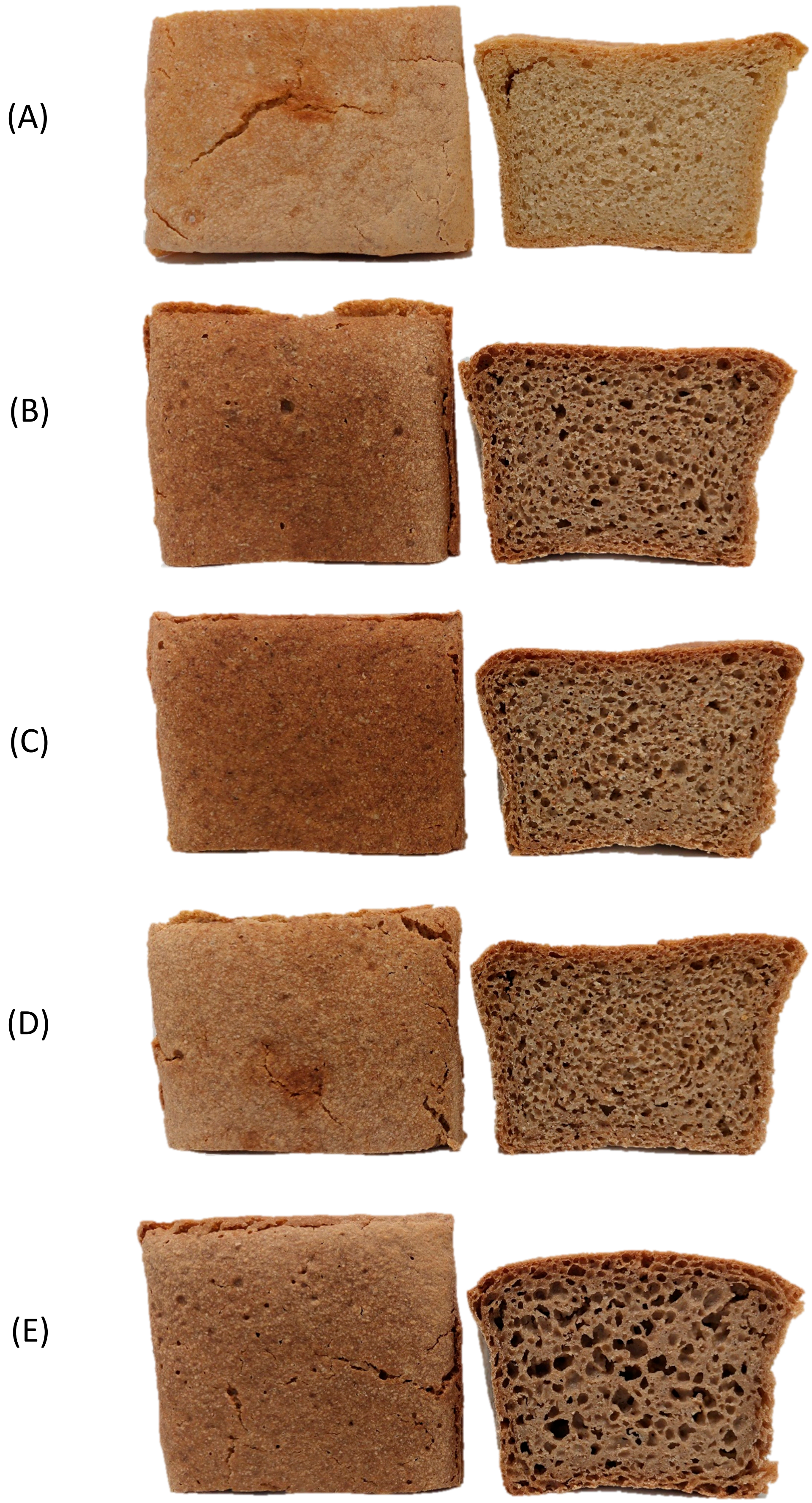

Supplement: Supplementary file 1 [file antioxidants-11-02022-s001.zip › antioxidants-1955914-supplementary.pdf]
